# Supplementary material for: Influence of microbiota-associated metabolic reprogramming on clinical outcome in patients with melanoma from the randomized adjuvant dendritic cell-based MIND-DC trial
Source: Nat Commun. 2024 Feb 23;15:1633. doi: 10.1038/s41467-024-45357-1 (PMC10891084; doi:10.1038/s41467-024-45357-1)
Supplement: Supplementary file 1 — Supplementary Information [file 41467_2024_45357_MOESM1_ESM.pdf]

MIND-DC manuscript, Alves Costa Silva et al.

Supplementary Figures

Figure S1

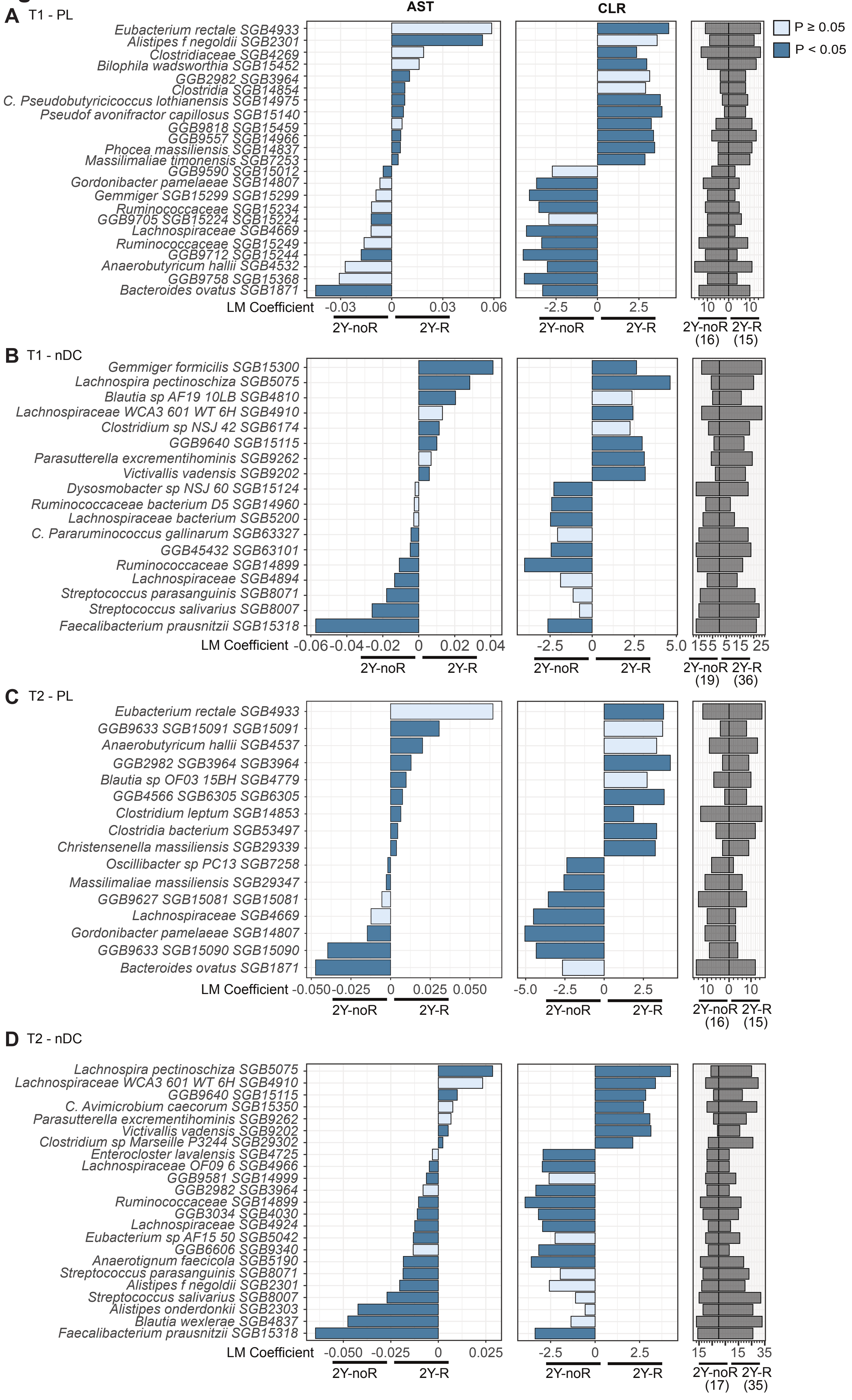

A T1 - PL
AST
CLR
P ≥ 0.05
P < 0.05
Eubacterium rectale SGB4933
Alistipes f negoldii SGB2301
Clostridiaceae SGB4269
Bilophila wadsworthia SGB15452
GGB2982 SGB3964
Clostridia SGB14854
C. Pseudobutyricicoccus lothianensis SGB14975
Pseudof avonifractor capillosus SGB15140
GGB9818 SGB15459
GGB9557 SGB14966
Phocea massiliensis SGB14837
Massilimaliae timonensis SGB7253
GGB9590 SGB15012
Gordonibacter pamelaeae SGB14807
Gemmiger SGB15299 SGB15299
Ruminococcaceae SGB15234
GGB9705 SGB15224 SGB15224
Lachnospiraceae SGB4669
Ruminococcaceae SGB15249
GGB9712 SGB15244
Anaerobutyricum hallii SGB4532
GGB9758 SGB15368
Bacteroides ovatus SGB1871
LM Coefficient
2Y-noR
2Y-R
2Y-noR (16)
2Y-R (15)
B T1 - nDC
Gemmiger formicilis SGB15300
Lachnospira pectinoschiza SGB5075
Blautia sp AF19 10LB SGB4810
Lachnospiraceae WCA3 601 WT 6H SGB4910
Clostridium sp NSJ 42 SGB6174
GGB9640 SGB15115
Parasutterella excrementihominis SGB9262
Victivallis vadensis SGB9202
Dysosmobacter sp NSJ 60 SGB15124
Ruminococcaceae bacterium D5 SGB14960
Lachnospiraceae bacterium SGB5200
C. Pararuminococcus gallinarum SGB63327
GGB45432 SGB63101
Ruminococcaceae SGB14899
Lachnospiraceae SGB4894
Streptococcus parasanguinis SGB8071
Streptococcus salivarius SGB8007
Faecalibacterium prausnitzii SGB15318
2Y-noR (19)
2Y-R (36)
C T2 - PL
Eubacterium rectale SGB4933
GGB9633 SGB15091 SGB15091
Anaerobutyricum hallii SGB4537
GGB2982 SGB3964 SGB3964
Blautia sp OF03 15BH SGB4779
GGB4566 SGB6305 SGB6305
Clostridium leptum SGB14853
Clostridia bacterium SGB53497
Christensenella massiliensis SGB29339
Oscillibacter sp PC13 SGB7258
Massilimaliae massiliensis SGB29347
GGB9627 SGB15081 SGB15081
Lachnospiraceae SGB4669
Gordonibacter pamelaeae SGB14807
GGB9633 SGB15090 SGB15090
Bacteroides ovatus SGB1871
2Y-noR (16)
2Y-R (15)
D T2 - nDC
Lachnospira pectinoschiza SGB5075
Lachnospiraceae WCA3 601 WT 6H SGB4910
GGB9640 SGB15115
C. Avimicrobium caecorum SGB15350
Parasutterella excrementihominis SGB9262
Victivallis vadensis SGB9202
Clostridium sp Marseille P3244 SGB29302
Enterocloster lavalensis SGB4725
Lachnospiraceae OF09 6 SGB4966
GGB9581 SGB14999
GGB2982 SGB3964
Ruminococcaceae SGB14899
GGB3034 SGB4030
Lachnospiraceae SGB4924
Eubacterium sp AF15 50 SGB5042
GGB6606 SGB9340
Anaerotignum faecicola SGB5190
Streptococcus parasanguinis SGB8071
Alistipes f negoldii SGB2301
Streptococcus salivarius SGB8007
Alistipes onderdonkii SGB2303
Blautia wexlerae SGB4837
Faecalibacterium prausnitzii SGB15318
2Y-noR (17)
2Y-R (35)

Supplementary Figure 1. Metagenomics analysis of MIND-DC patient stools according to 2Y-R for each treatment arm at 2 time points.

A-D. Differential abundance analysis for SGBs performed between recurrence at 2 years (2Y-R) and no recurrence at 2 years (2Y-noR) for patients at T1 in the placebo (PL, A) or in the natural dendritic cell (nDC, B) arms and at T2 in the PL (C) or in the nDC (D) arms. In each panel, first and second barplot on the left report the linear model coefficients (MaAsLin2 coefficient) for microbial species-level genome bins (SGBs) that are found associated either after arcsine square root (arcsin-sqrt) transformation (AST, left columns) or centered-log-ratio (CLR, middle columns) transformation with $p<0.05$ (no association presented Benjamini-Hochberg $Q<0.2$). Barplots on the right report the number of samples in which each SGB was present (with direction to the left for 2Y-noR and with direction to the right for 2Y-R) in the corresponding group. The numbers of patients considered in this analysis and microbial prevalence are detailed in Supplementary Table 2 and Supplementary Data file 2, respectively. Source data are provided as a Source Data file.

Figure S2

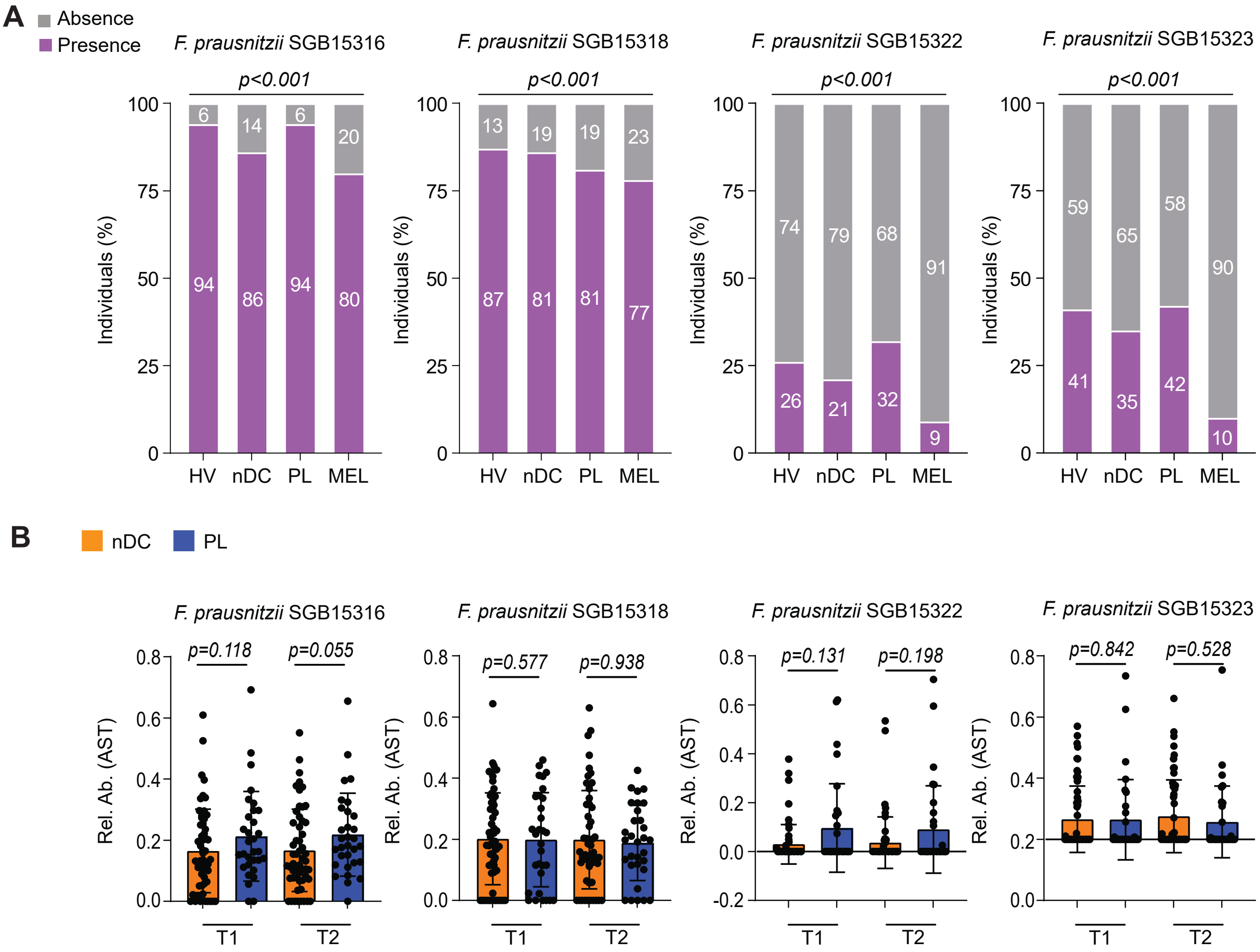
A
Absence
Presence
F. prausnitzii SGB15316
F. prausnitzii SGB15318
F. prausnitzii SGB15322
F. prausnitzii SGB15323
p<0.001
Individuals (%)
0
25
50
75
100
HV
nDC
PL
MEL
6
94
14
86
6
94
20
80
13
87
19
81
19
81
23
77
74
26
79
21
68
32
91
9
59
41
65
35
58
42
90
10
B
nDC
PL
F. prausnitzii SGB15316
F. prausnitzii SGB15318
F. prausnitzii SGB15322
F. prausnitzii SGB15323
Rel. Ab. (AST)
p=0.118
p=0.055
p=0.577
p=0.938
p=0.131
p=0.198
p=0.842
p=0.528
0.0
0.2
0.4
0.6
0.8
-0.2
T1
T2

Supplementary Figure 2. Relative abundances of distinct bacterial species associated with recurrence or survival including species-level genome bin (SGBs) of *Faecalibacterium prausnitzii.*

A. Prevalence of *Faecalibacterium prausnitzii* SGB15316, SGB15318, SGB15322 and SGB15323 between healthy volunteers (HV, n=6048), publicly available patients with melanoma (n=1036) together with patients into MIND-DC trial (MEL, n=1124 in total), placebo (PL, n=31) and natural dendritic cell (nDC, n=57) arms from MIND-DC. B. Boxplots showing relative abundances of distinct *F. prausnitzii* SGBs according to treatment arms at T1 (left, N=88) and T2 (right, n=85). All scatter dot plots indicate the mean with SD. The range of outliers is depicted by whiskers. The p-values are related to the group comparison using the Mann–Whitney test. Source data are provided as a Source Data file.

Figure S3

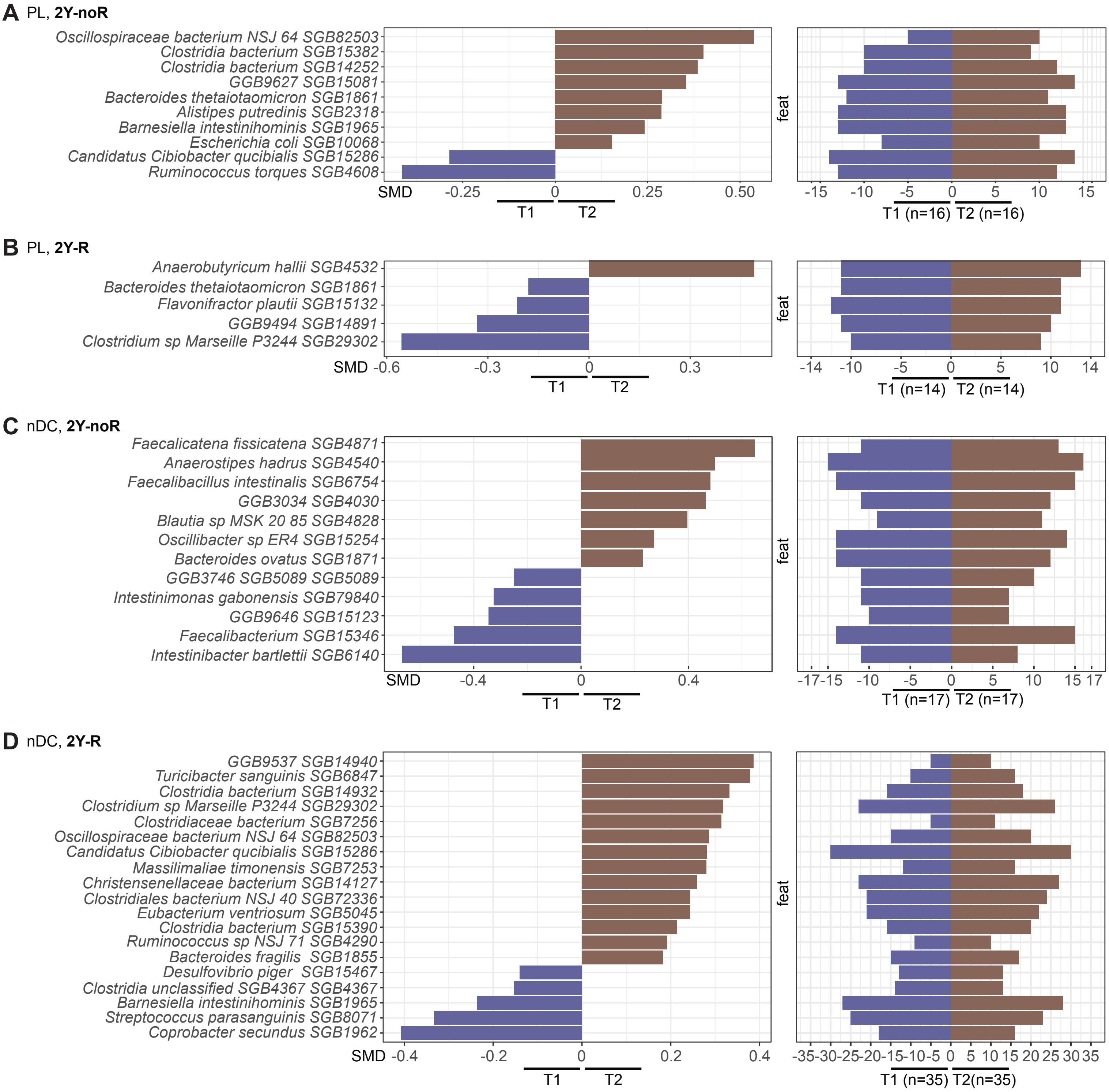

A PL, 2Y-noR
Oscillospiraceae bacterium NSJ 64 SGB82503
Clostridia bacterium SGB15382
Clostridia bacterium SGB14252
GGB9627 SGB15081
Bacteroides thetaiotaomicron SGB1861
Alistipes putredinis SGB2318
Barnesiella intestinihominis SGB1965
Escherichia coli SGB10068
Candidatus Cibiobacter qucibialis SGB15286
Ruminococcus torques SGB4608
SMD -0.25 0 0.25 0.50
T1 T2
feat
-15 -10 -5 0 5 10 15
T1 (n=16) T2 (n=16)
B PL, 2Y-R
Anaerobutyricum hallii SGB4532
Bacteroides thetaiotaomicron SGB1861
Flavonifractor plautii SGB15132
GGB9494 SGB14891
Clostridium sp Marseille P3244 SGB29302
SMD -0.6 -0.3 0 0.3
T1 T2
feat
-14 -10 -5 0 5 10 14
T1 (n=14) T2 (n=14)
C nDC, 2Y-noR
Faecalicatena fissicatena SGB4871
Anaerostipes hadrus SGB4540
Faecalibacillus intestinalis SGB6754
GGB3034 SGB4030
Blautia sp MSK 20 85 SGB4828
Oscillibacter sp ER4 SGB15254
Bacteroides ovatus SGB1871
GGB3746 SGB5089 SGB5089
Intestinimonas gabonensis SGB79840
GGB9646 SGB15123
Faecalibacterium SGB15346
Intestinibacter bartlettii SGB6140
SMD -0.4 0 0.4
T1 T2
feat
-17-15 -10 -5 0 5 10 15 17
T1 (n=17) T2 (n=17)
D nDC, 2Y-R
GGB9537 SGB14940
Turicibacter sanguinis SGB6847
Clostridia bacterium SGB14932
Clostridium sp Marseille P3244 SGB29302
Clostridiaceae bacterium SGB7256
Oscillospiraceae bacterium NSJ 64 SGB82503
Candidatus Cibiobacter qucibialis SGB15286
Massilimaliae timonensis SGB7253
Christensenellaceae bacterium SGB14127
Clostridiales bacterium NSJ 40 SGB72336
Eubacterium ventriosum SGB5045
Clostridia bacterium SGB15390
Ruminococcus sp NSJ 71 SGB4290
Bacteroides fragilis SGB1855
Desulfovibrio piger SGB15467
Clostridia unclassified SGB4367 SGB4367
Barnesiella intestinihominis SGB1965
Streptococcus parasanguinis SGB8071
Coprobacter secundus SGB1962
SMD -0.4 -0.2 0 0.2 0.4
T1 T2
feat
-35-30-25-20-15-10-5 0 5 10 15 20 25 30 35
T1 (n=35) T2(n=35)

Supplementary Figure 3. Metagenomics-based differences between T1 and T2 according to treatment arms.

A-D. Differential abundance analysis between T1 and T2 according to treatment arm and recurrence at 2 years performed via a Wilcoxon signed-rank test on arcsine square root (arcsin-sqrt) transformation (AST) transformed relative abundances in patients included in the placebo arm (PL) with no recurrence at 2 years (2Y-noR, A) or recurrence at 2 years (2Y-R, B), and in patients included in the natural dendritic cell arm (nDC) with 2y-noR (C) or 2Y-R (D). Associations with $P < 0.05$ are reported (no association presented Benjamini-Hochberg $Q<0.2$). In each panel, barplots on the left report the standardized mean difference (scale on the x-axis) between the two time points for each microbiome feature, while the barplots on the right report the number of positive samples for each microbiome feature at the two time points. SMD: standardized mean difference. Source data are provided as a Source Data file.

Figure S4

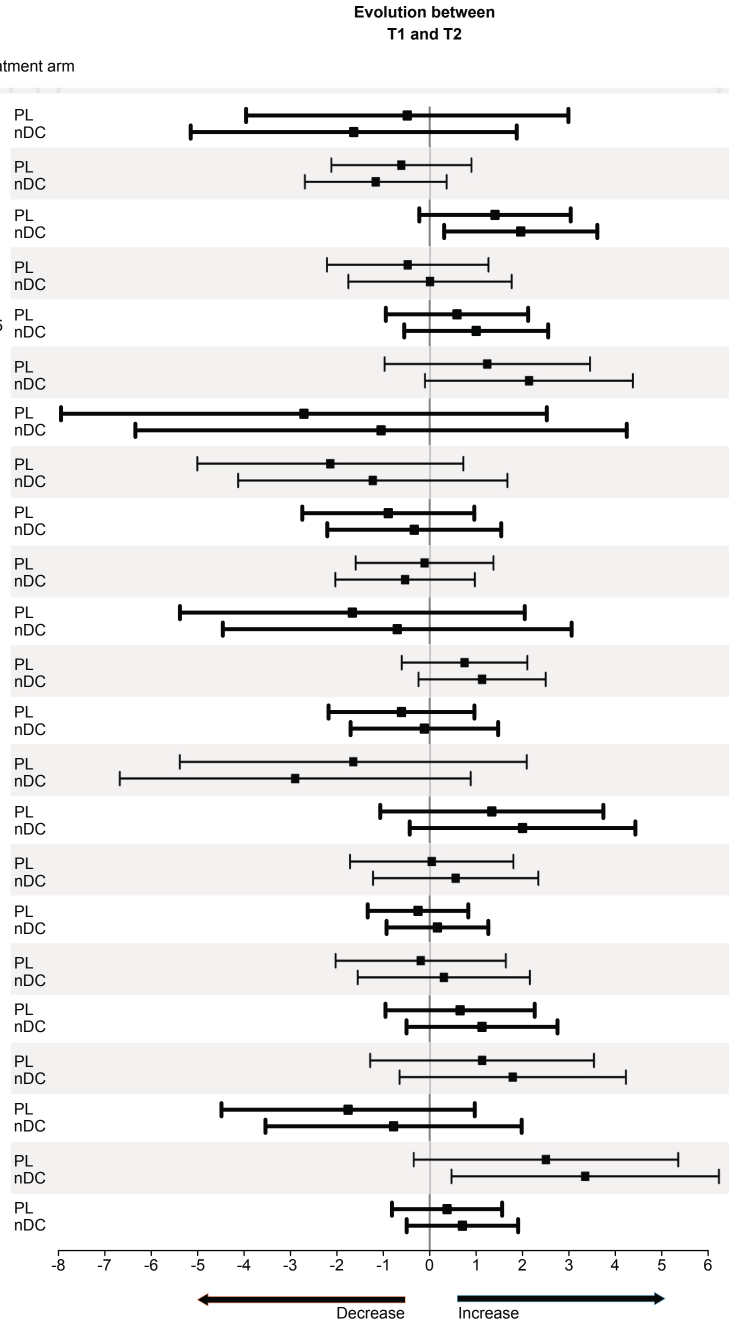

Metagenomics species	Treatment arm	Evolution statistically different from 0 (p-value)	Different evolution between the 2 arms? (p-value)
Actinomyces sp ICM47 SGB17167	PL	0.7822	0.0107
	nDC	0.3562	
Barnesiella intestinihominis SGB1965	PL	0.4180	0.0052
	nDC	0.1316	
Blautia sp MSK 20 85 SGB4828	PL	0.0900	0.0091
	nDC	0.0205	
Candidatus Cibiobacter qucibialis SGB15286	PL	0.5849	0.0332
	nDC	1.0000	
Candidatus Pseudobutyricicoccus lothianensis SGB14975	PL	0.4476	0.0367
	nDC	0.2022	
Christensenella massiliensis SGB29339	PL	0.2703	0.0020
	nDC	0.0616	
Christensenellaceae bacterium NSJ 53 SGB82545	PL	0.3057	0.0145
	nDC	0.6946	
Clostridia bacterium SGB15401	PL	0.1400	0.0142
	nDC	0.4000	
Clostridia bacterium SGB5180	PL	0.3406	0.0200
	nDC	0.7244	
Clostridiaceae unclassified SGB4769	PL	0.8769	0.0292
	nDC	0.4803	
Clostridium sp Marseille P3244 SGB29302	PL	0.3745	0.0439
	nDC	0.7118	
Collinsella aerofaciens SGB14546	PL	0.2771	0.0310
	nDC	0.1069	
Dorea longicatena SGB4582	PL	0.4434	0.0157
	nDC	0.8860	
Enterocloster citroniae SGB4761	PL	0.3816	0.0101
	nDC	0.1303	
Eubacteriaceae bacterium SGB3958	PL	0.2711	0.0331
	nDC	0.1054	
Eubacterium ventriosum SGB5045	PL	0.9664	0.0238
	nDC	0.5380	
GGB34228 SGB72916	PL	0.6445	0.0032
	nDC	0.7630	
GGB45432 SGB63101	PL	0.8291	0.0358
	nDC	0.7498	
GGB9522 SGB14921	PL	0.4200	0.0234
	nDC	0.1717	
Methanobrevibacter smithii SGB714	PL	0.3564	0.0342
	nDC	0.1494	
Ruminococcaceae bacterium SGB15272	PL	0.2034	0.0060
	nDC	0.5760	
Ruminococcus torques SGB4608	PL	0.0847	0.0219
	nDC	0.0235	
Senegalimassilia anaerobia SGB14824	PL	0.5327	0.0312
	nDC	0.2462	

Supplementary Figure 4. Natural dendritic cells (nDC)-induced modulations of metagenomics (MG) overtime.

Short list of the statistically significant (without FDR correction) MG species differential evolution between the treatment arms nDC (n=54) versus placebo (PL, n=30), modeled using linear regression adjusted for the age, gender, melanoma stage, ECOG PS, and BMI. The square represents the point estimate of the evolution of the MGB between T1 and T2, and the horizontal segments represent their 95% confidence intervals. The first column of p-values is related to the Wald test that the evolution is different from 0. The last column of p-values is related to the Wald test of the difference of the evolution between the two arms.

Figure S5

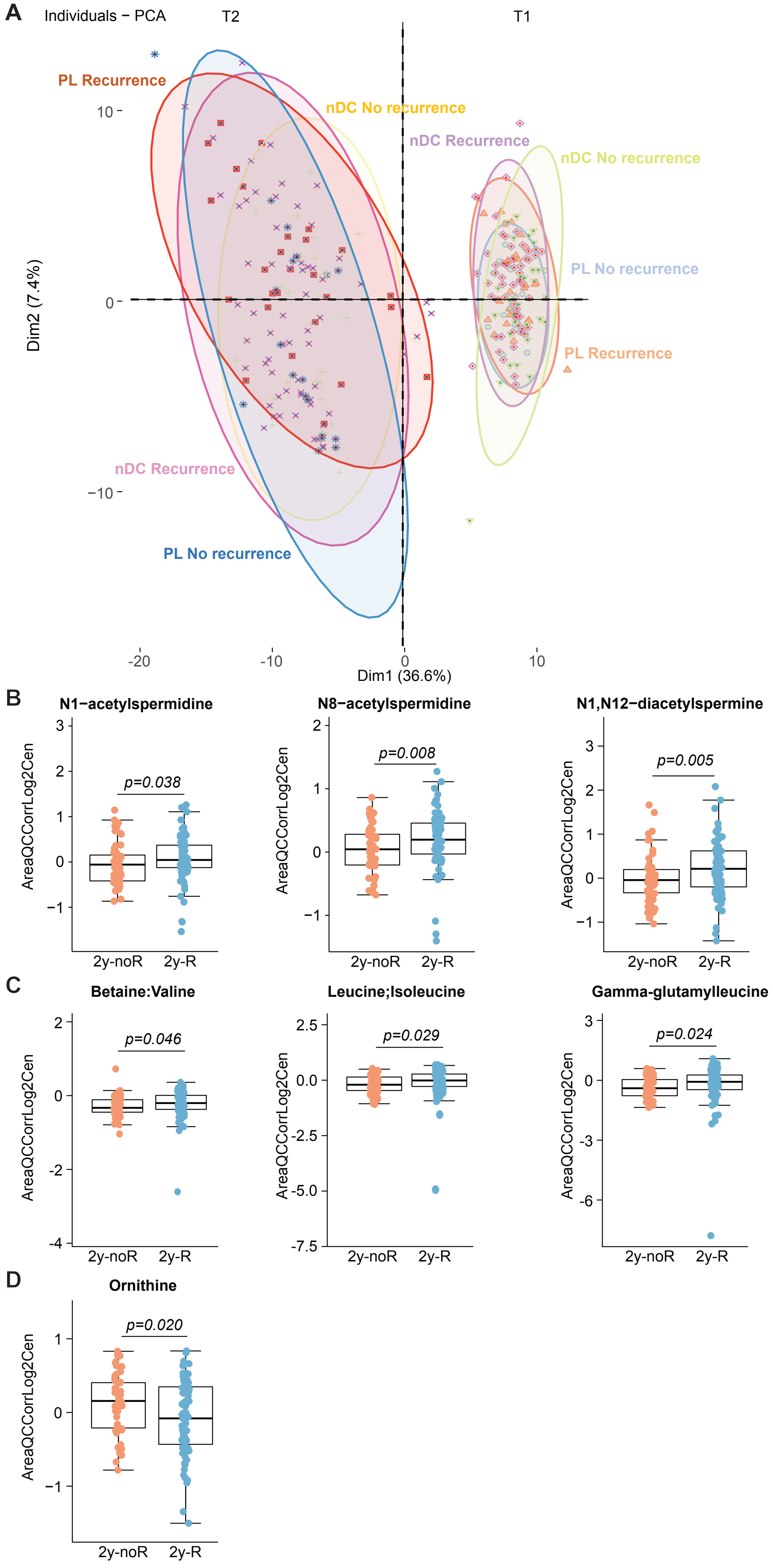
A
Individuals − PCA
T2
T1
PL Recurrence
nDC No recurrence
nDC Recurrence
nDC No recurrence
PL No recurrence
PL Recurrence
nDC Recurrence
PL No recurrence
Dim2 (7.4%)
Dim1 (36.6%)
B
N1−acetylspermidine
p=0.038
N8−acetylspermidine
p=0.008
N1,N12−diacetylspermine
p=0.005
C
Betaine:Valine
p=0.046
Leucine;Isoleucine
p=0.029
Gamma-glutamylleucine
p=0.024
D
Ornithine
p=0.020
AreaQCCorrLog2Cen
2y-noR
2y-R

Supplementary Figure 5. Metabolic modulations during therapy.

A. Principal component analysis (PCA) plots representing the circulating metabolome according to treatment arms natural dendritic cell (nDC, n=95) versus placebo (PL, n=49), recurrence or no recurrence at 2 years (2Y-R or 2Y-noR, respectively), and time points baseline (T1, right) or after 2 bimonthly injections (T2, left). Blue circle: PL 2Y-noR at T1; Orange triangle: 2Y-noR at T2; Yellow crosses: nDC 2Y-noR at T2; Pink cross: nDC 2Y-R at T2; Purple diamond: nDC 2Y-R at T1; Green upside triangle: nDC 2Y-noR at T1; Red crossed square: PL 2Y-R at T2; Blue asterisk: PL 2Y-noR at T2. B-D. Boxplots showing the relative abundances of metabolites according to 2Y-R (n=83) or 2Y-noR (n=56). All boxplot indicates the interquartile range Q1 to Q3 with Q2 (median) in the center. The range of outliers is depicted by whiskers. The p-values are related to the group comparison using the Mann–Whitney test. Source data are provided as a Source Data file.

Figure S6

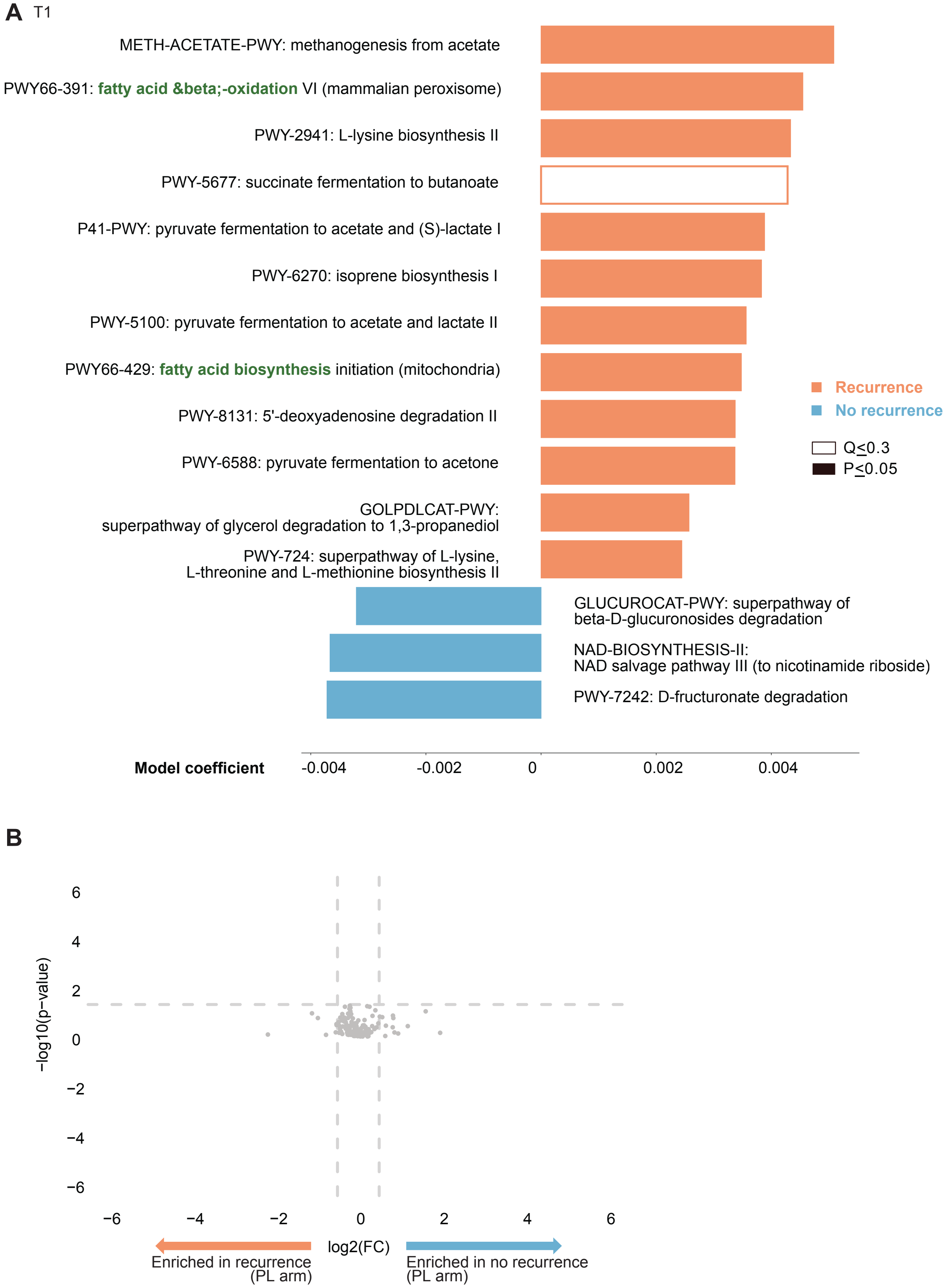
A T1
METH-ACETATE-PWY: methanogenesis from acetate
PWY66-391: fatty acid β-oxidation VI (mammalian peroxisome)
PWY-2941: L-lysine biosynthesis II
PWY-5677: succinate fermentation to butanoate
P41-PWY: pyruvate fermentation to acetate and (S)-lactate I
PWY-6270: isoprene biosynthesis I
PWY-5100: pyruvate fermentation to acetate and lactate II
PWY66-429: fatty acid biosynthesis initiation (mitochondria)
PWY-8131: 5'-deoxyadenosine degradation II
PWY-6588: pyruvate fermentation to acetone
GOLPDLCAT-PWY: superpathway of glycerol degradation to 1,3-propanediol
PWY-724: superpathway of L-lysine, L-threonine and L-methionine biosynthesis II
GLUCUROCAT-PWY: superpathway of beta-D-glucuronosides degradation
NAD-BIOSYNTHESIS-II: NAD salvage pathway III (to nicotinamide riboside)
PWY-7242: D-fructuronate degradation
Recurrence
No recurrence
Q≤0.3
P≤0.05
Model coefficient
-0.004
-0.002
0
0.002
0.004
B
−log10(p−value)
6
4
2
0
−2
−4
−6
−6
−4
−2
0
2
4
6
log2(FC)
Enriched in recurrence (PL arm)
Enriched in no recurrence (PL arm)

Supplementary Figure 6. Associations of functional gene pathways with overall recurrence.
A. Linear model coefficients for the comparisons in overall recurrence at baseline (T1, n=86) for microbial gene functional pathways corrected for age, gender and treatment. Adjusted P-values (Q) are computed via the Benjamini-Hochberg procedure. Any association with either p<=0.05 or Q<=0.3 has been selected. Source data are provided as a Source Data file. B. Volcano-plots based on metabolomics data highlighting no significant difference between recurrence (left side, orange) and no recurrence (right side, blue) at T1 in placebo (PL, n=49) arm. X-axis: log2 fold change of metabolites; Y-axis: fold change of –log10 P value determined by the Mann–Whitney test. Source data are provided as a Source Data file.

Figure S7

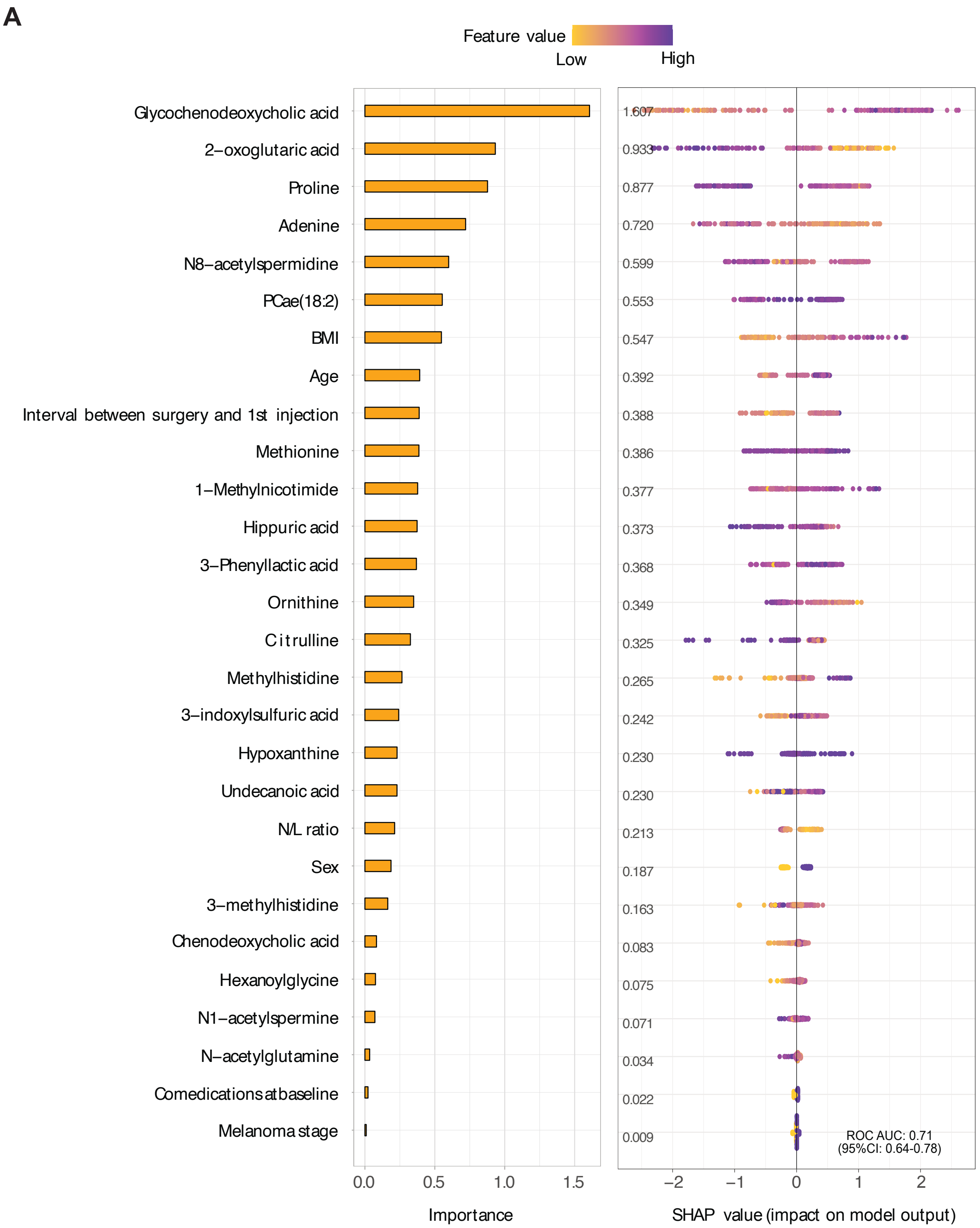
A
Feature value
Low
High
Glycochenodeoxycholic acid
2–oxoglutaric acid
Proline
Adenine
N8–acetylspermidine
PCae(18:2)
BMI
Age
Interval between surgery and 1st injection
Methionine
1–Methylnicotimide
Hippuric acid
3–Phenyllactic acid
Ornithine
Citrulline
Methylhistidine
3–indoxylsulfuric acid
Hypoxanthine
Undecanoic acid
N/L ratio
Sex
3–methylhistidine
Chenodeoxycholic acid
Hexanoylglycine
N1–acetylspermine
N–acetylglutamine
Comedications at baseline
Melanoma stage
0.0
0.5
1.0
1.5
Importance
1.607
0.933
0.877
0.720
0.599
0.553
0.547
0.392
0.388
0.386
0.377
0.373
0.368
0.349
0.325
0.265
0.242
0.230
0.230
0.213
0.187
0.163
0.083
0.075
0.071
0.034
0.022
0.009
ROC AUC: 0.71
(95%CI: 0.64-0.78)
−2
−1
0
1
2
SHAP value (impact on model output)

Supplementary Figure 7. Identification of the most representative metabolites differentiating the two treatment arms by machine learning approach.

A feature selection was conducted using Boruta feature selection algorithm based on XGboost to identify most relevant features in the metabolomics analysis at T1 (n=143). Selected features ranked by importance in the prediction of treatment arm attribution: natural dendritic cell (nDC, n=95) versus placebo (PL, n=49) arm status (left panel). Features are clinical parameters and 152 metabolites. SHapley Additive exPlanations (SHAP) values for each feature per patient are positive when the value of the feature increases the prediction of nDC randomization, negative otherwise (right panel). Each dot represents one patient and the color depicts the value of each feature. Source data are provided as a Source Data file.

Figure S8

A

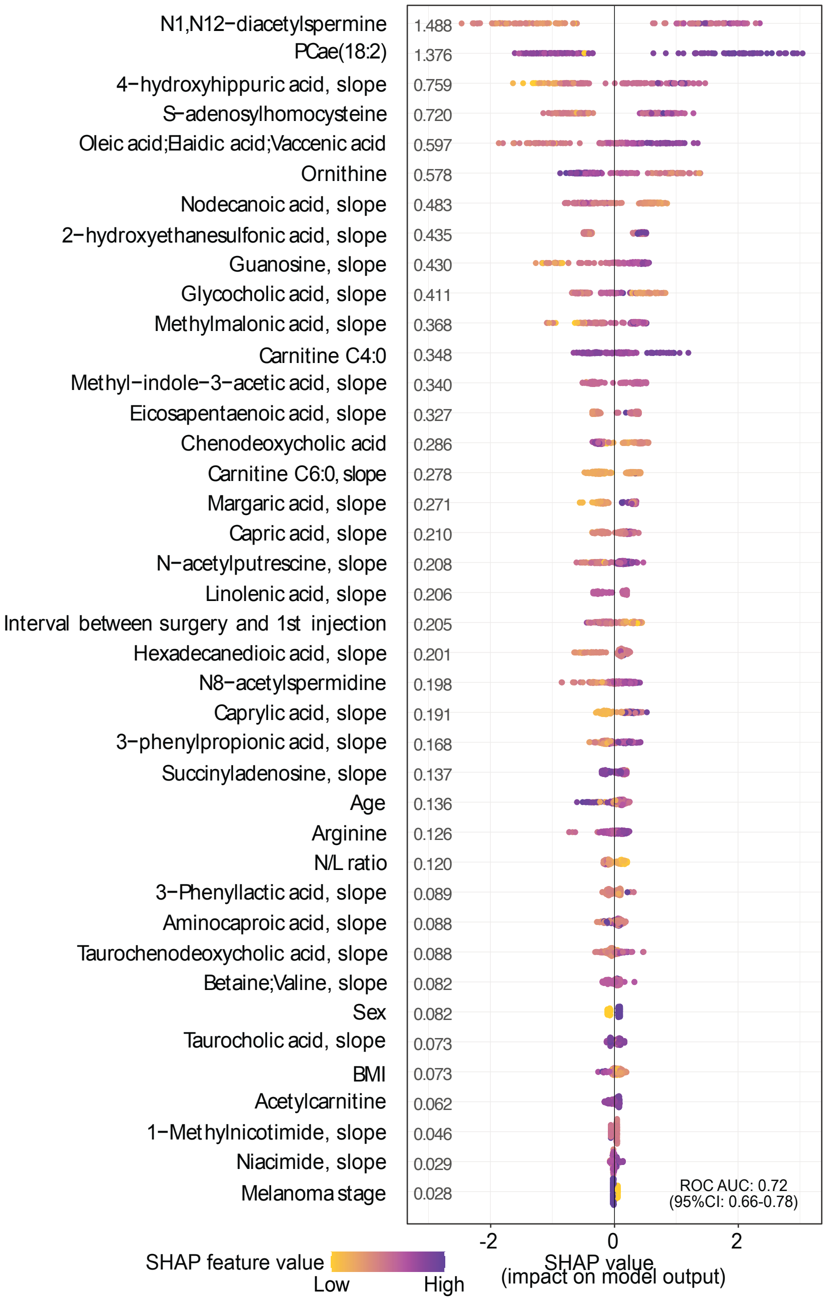

N1,N12−diacetylspermine
PCae(18:2)
4−hydroxyhippuric acid, slope
S−adenosylhomocysteine
Oleic acid;Elaidic acid;Vaccenic acid
Ornithine
Nodecanoic acid, slope
2−hydroxyethanesulfonic acid, slope
Guanosine, slope
Glycocholic acid, slope
Methylmalonic acid, slope
Carnitine C4:0
Methyl−indole−3−acetic acid, slope
Eicosapentaenoic acid, slope
Chenodeoxycholic acid
Carnitine C6:0, slope
Margaric acid, slope
Capric acid, slope
N−acetylputrescine, slope
Linolenic acid, slope
Interval between surgery and 1st injection
Hexadecanedioic acid, slope
N8−acetylspermidine
Caprylic acid, slope
3−phenylpropionic acid, slope
Succinyladenosine, slope
Age
Arginine
N/L ratio
3−Phenyllactic acid, slope
Aminocaproic acid, slope
Taurochenodeoxycholic acid, slope
Betaine;Valine, slope
Sex
Taurocholic acid, slope
BMI
Acetylcarnitine
1−Methylnicotimide, slope
Niacimide, slope
Melanoma stage
1.488
1.376
0.759
0.720
0.597
0.578
0.483
0.435
0.430
0.411
0.368
0.348
0.340
0.327
0.286
0.278
0.271
0.210
0.208
0.206
0.205
0.201
0.198
0.191
0.168
0.137
0.136
0.126
0.120
0.089
0.088
0.088
0.082
0.082
0.073
0.073
0.062
0.046
0.029
0.028
ROC AUC: 0.72
(95%CI: 0.66-0.78)
-2
0
2
SHAP feature value
Low
High
SHAP value
(impact on model output)

B

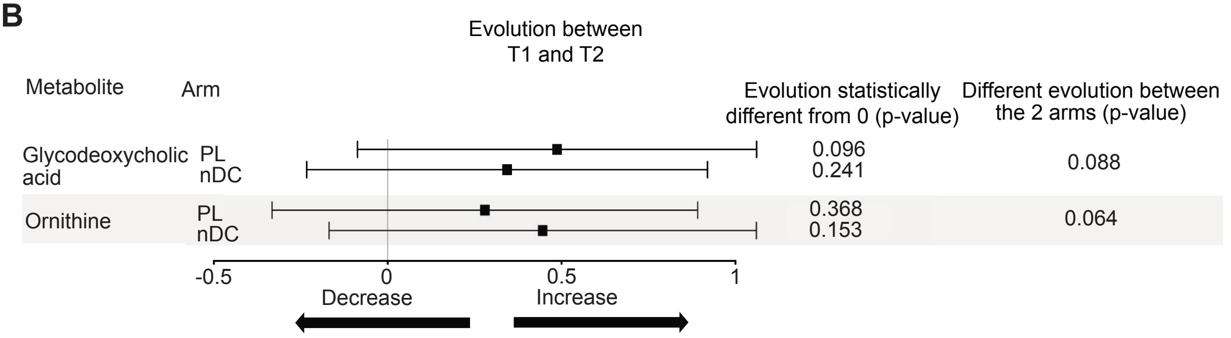

Evolution between
T1 and T2
Metabolite
Arm
Evolution statistically
different from 0 (p-value)
Different evolution between
the 2 arms (p-value)
Glycodeoxycholic acid
PL
nDC
0.096
0.241
0.088
Ornithine
PL
nDC
0.368
0.153
0.064
-0.5
0
0.5
1
Decrease
Increase

Supplementary Figure 8. Identification of the most relevant baseline values and trajectory of metagenomics (MG) and metabolomics (MB) for the prediction of the recurrence at 2 years (2Y-R).

A. A feature selection was conducted using Boruta feature selection algorithm based on XGboost to identify most relevant feature evolution in between T1 and T2 in the MB analysis (n=143). Selected features ranked by importance in the prediction of treatment arm attribution (2Y-R versus 2Y-noR) status, pooling the two treatment arms. Features are clinical parameters, and 152 metabolites. SHapley Additive exPlanations (SHAP) values for each feature per patient are positive when the value of the feature increases the prediction of 2Y-R, negative otherwise. Each dot represents one patient and the color depicts the value of each feature. Source data are provided as a Source Data file. AUC: Area Under the ROC Curve; ROC: Receiver Operating Characteristic. B. Short list of the statistically significant without False Discovery Rate (FDR) correction metabolite differential evolution between the treatment arm natural dendritic cell (nDC) versus placebo (PL), modeled using linear regression adjusted for the age, gender, melanoma stage, ECOG-PS, and body mass index (BMI). The square represents the point estimate of the evolution of the metabolite between T1 and T2, and the horizontal segments represent their 95% confidence intervals. The first column of p-values is related to the Wald test that the evolution is different from 0. The last column of p-values is related to the Wald test of the difference of the evolution between the two arms.

Figure S9

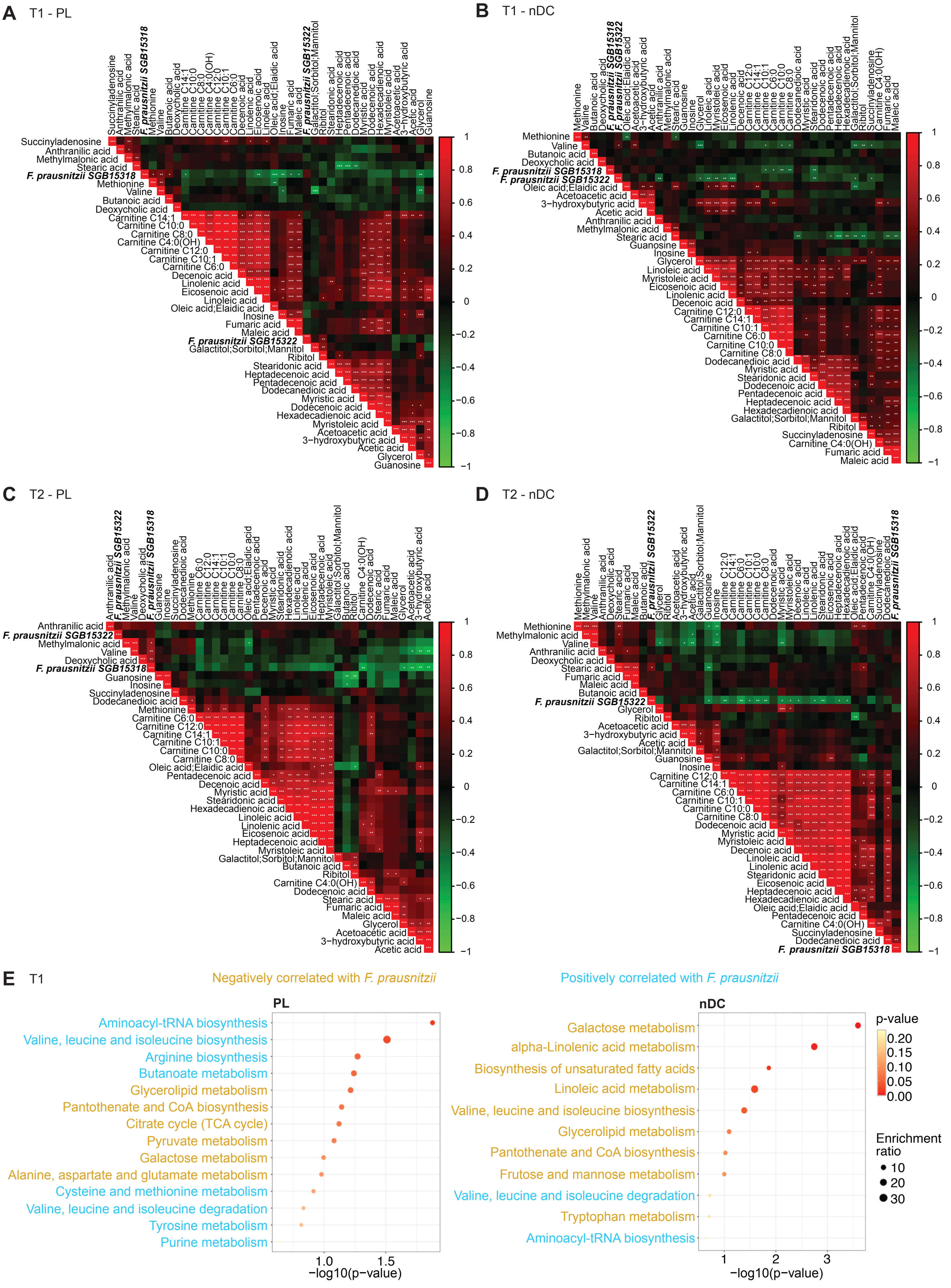
A
T1 - PL
Succinyladenosine
Anthranilic acid
Methylmalonic acid
Stearic acid
F. prausnitzii SGB15318
Methionine
Valine
Butanoic acid
Deoxycholic acid
Carnitine C14:1
Carnitine C10:0
Carnitine C8:0
Carnitine C4:0(OH)
Carnitine C12:0
Carnitine C10:1
Carnitine C6:0
Decenoic acid
Linolenic acid
Eicosenoic acid
Linoleic acid
Oleic acid;Elaidic acid
Inosine
Fumaric acid
Maleic acid
F. prausnitzii SGB15322
Galactitol;Sorbitol;Mannitol
Ribitol
Stearidonic acid
Heptadecenoic acid
Pentadecenoic acid
Dodecanedioic acid
Myristic acid
Dodecenoic acid
Hexadecadienoic acid
Myristoleic acid
Acetoacetic acid
3−hydroxybutyric acid
Acetic acid
Glycerol
Guanosine
1
0.8
0.6
0.4
0.2
0
−0.2
−0.4
−0.6
−0.8
−1
B
T1 - nDC
Methionine
Valine
Butanoic acid
Deoxycholic acid
F. prausnitzii SGB15318
F. prausnitzii SGB15322
Oleic acid;Elaidic acid
Acetoacetic acid
3−hydroxybutyric acid
Acetic acid
Anthranilic acid
Methylmalonic acid
Stearic acid
Guanosine
Inosine
Glycerol
Linoleic acid
Myristoleic acid
Eicosenoic acid
Linolenic acid
Decenoic acid
Carnitine C12:0
Carnitine C14:1
Carnitine C10:1
Carnitine C6:0
Carnitine C10:0
Carnitine C8:0
Dodecanedioic acid
Myristic acid
Stearidonic acid
Dodecenoic acid
Pentadecenoic acid
Heptadecenoic acid
Hexadecadienoic acid
Galactitol;Sorbitol;Mannitol
Ribitol
Succinyladenosine
Carnitine C4:0(OH)
Fumaric acid
Maleic acid
C
T2 - PL
Anthranilic acid
F. prausnitzii SGB15322
Methylmalonic acid
Valine
Deoxycholic acid
F. prausnitzii SGB15318
Guanosine
Inosine
Succinyladenosine
Dodecanedioic acid
Methionine
Carnitine C6:0
Carnitine C12:0
Carnitine C14:1
Carnitine C10:1
Carnitine C10:0
Carnitine C8:0
Oleic acid;Elaidic acid
Pentadecenoic acid
Decenoic acid
Myristic acid
Stearidonic acid
Hexadecadienoic acid
Linoleic acid
Linolenic acid
Eicosenoic acid
Heptadecenoic acid
Myristoleic acid
Galactitol;Sorbitol;Mannitol
Butanoic acid
Ribitol
Carnitine C4:0(OH)
Dodecenoic acid
Stearic acid
Fumaric acid
Maleic acid
Glycerol
Acetoacetic acid
3−hydroxybutyric acid
Acetic acid
D
T2 - nDC
Methionine
Methylmalonic acid
Valine
Anthranilic acid
Deoxycholic acid
Stearic acid
Fumaric acid
Maleic acid
Butanoic acid
F. prausnitzii SGB15322
Glycerol
Ribitol
Acetoacetic acid
3−hydroxybutyric acid
Acetic acid
Galactitol;Sorbitol;Mannitol
Guanosine
Inosine
Carnitine C12:0
Carnitine C14:1
Carnitine C6:0
Carnitine C10:1
Carnitine C10:0
Carnitine C8:0
Dodecenoic acid
Myristic acid
Myristoleic acid
Decenoic acid
Linoleic acid
Linolenic acid
Stearidonic acid
Eicosenoic acid
Heptadecenoic acid
Hexadecadienoic acid
Oleic acid;Elaidic acid
Pentadecenoic acid
Carnitine C4:0(OH)
Succinyladenosine
Dodecanedioic acid
F. prausnitzii SGB15318
E
T1
Negatively correlated with F. prausnitzii
Positively correlated with F. prausnitzii
PL
Aminoacyl-tRNA biosynthesis
Valine, leucine and isoleucine biosynthesis
Arginine biosynthesis
Butanoate metabolism
Glycerolipid metabolism
Pantothenate and CoA biosynthesis
Citrate cycle (TCA cycle)
Pyruvate metabolism
Galactose metabolism
Alanine, aspartate and glutamate metabolism
Cysteine and methionine metabolism
Valine, leucine and isoleucine degradation
Tyrosine metabolism
Purine metabolism
1.0
1.5
−log10(p−value)
nDC
Galactose metabolism
alpha-Linolenic acid metabolism
Biosynthesis of unsaturated fatty acids
Linoleic acid metabolism
Valine, leucine and isoleucine biosynthesis
Glycerolipid metabolism
Pantothenate and CoA biosynthesis
Frutose and mannose metabolism
Valine, leucine and isoleucine degradation
Tryptophan metabolism
Aminoacyl-tRNA biosynthesis
1
2
3
−log10(p−value)
p-value
0.20
0.15
0.10
0.05
0.00
Enrichment ratio
10
20
30

Supplementary Figure 9. Associations between *Faecalibacterium prausnitzii* a species-level genome bin (SGBs) 15318 and 15322 abundances with FA for each treatment arms at both time points.

A-D. Pearson correlation of concentration-fitted metabolites and *F. prausnitzii* SGB15318 and SGB15322 prevalence at T1 (A-B) and T2 (C-D) in placebo (PL, n=31, A and C) and natural dendritic cell (nDC, n=60, B and D) arms. Green color stands for inverse associations. Red color denotes positive associations. Symbols on the plot represent the level of significance denoting Pearson's correlation test significant associations at p value < 0.05. E. Metabolic pathway-enrichment analysis performed in MetaboAnalyst using a KEGG database of a set of metabolites found to be significantly correlated with *F. prausnitzii* SGB15318 and SGB15322 in patients into natural dendritic cell (nDC, n=60) and placebo (PL, n=31) arms at baseline (T1). Source data are provided as a Source Data file.

Figure S10

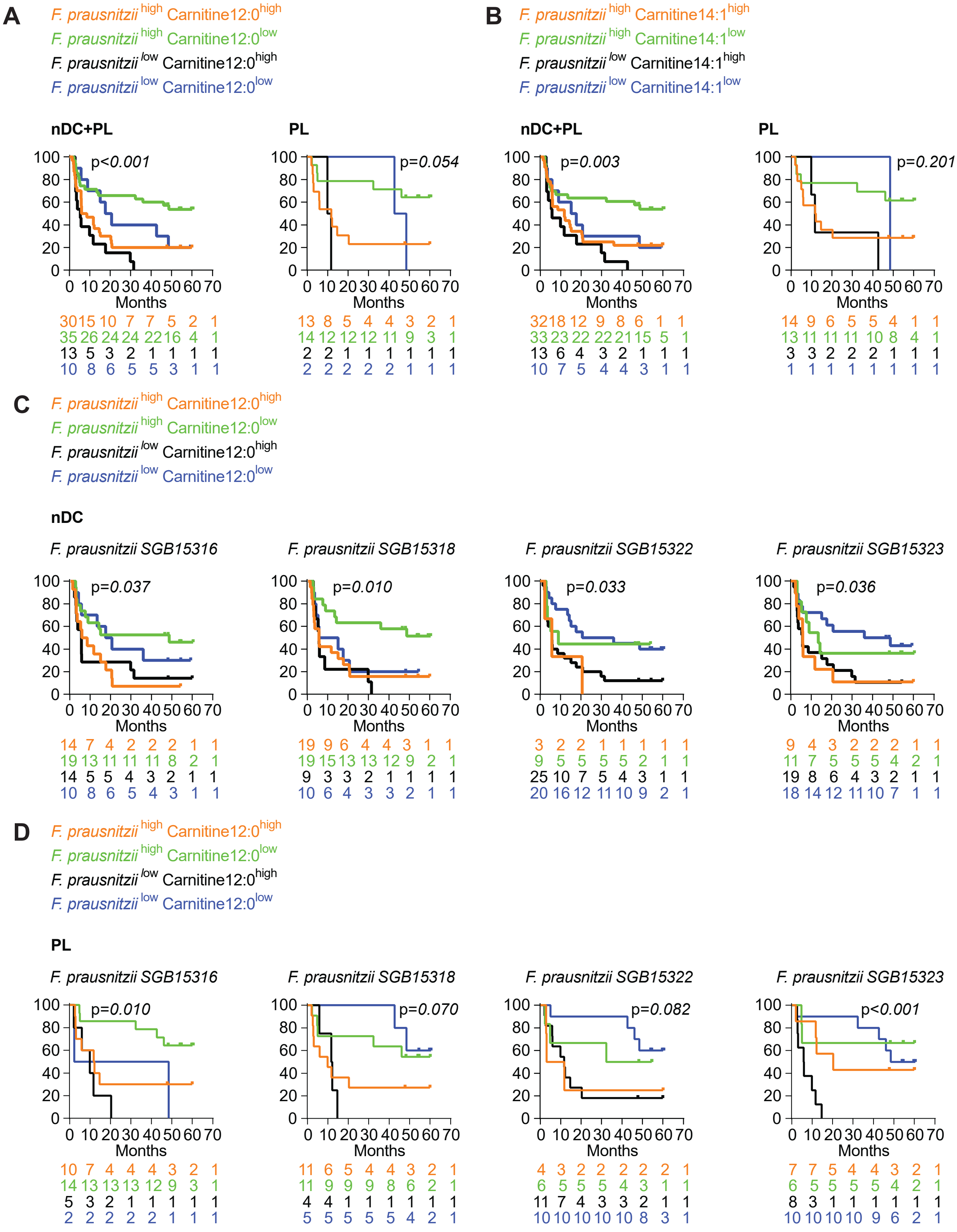

A
F. prausnitzii high Carnitine12:0 high
F. prausnitzii high Carnitine12:0 low
F. prausnitzii low Carnitine12:0 high
F. prausnitzii low Carnitine12:0 low
nDC+PL
p<0.001
Months
30 15 10 7 7 5 2 1
35 26 24 24 22 16 4 1
13 5 3 2 1 1 1 1
10 8 6 5 5 3 1 1
PL
p=0.054
Months
13 8 5 4 4 3 2 1
14 12 12 12 11 9 3 1
2 2 1 1 1 1 1 1
2 2 2 2 2 1 1 1
B
F. prausnitzii high Carnitine14:1 high
F. prausnitzii high Carnitine14:1 low
F. prausnitzii low Carnitine14:1 high
F. prausnitzii low Carnitine14:1 low
nDC+PL
p=0.003
Months
32 18 12 9 8 6 1 1
33 23 22 22 21 15 5 1
13 6 4 3 2 1 1 1
10 7 5 4 4 3 1 1
PL
p=0.201
Months
14 9 6 5 5 4 1 1
13 11 11 11 10 8 4 1
3 3 2 2 2 1 1 1
1 1 1 1 1 1 1 1
C
F. prausnitzii high Carnitine12:0 high
F. prausnitzii high Carnitine12:0 low
F. prausnitzii low Carnitine12:0 high
F. prausnitzii low Carnitine12:0 low
nDC
F. prausnitzii SGB15316
p=0.037
Months
14 7 4 2 2 2 1 1
19 13 11 11 11 8 2 1
14 5 5 4 3 2 1 1
10 8 6 5 4 3 1 1
F. prausnitzii SGB15318
p=0.010
Months
19 9 6 4 4 3 1 1
19 15 13 13 12 9 2 1
9 3 3 2 1 1 1 1
10 6 4 3 3 2 1 1
F. prausnitzii SGB15322
p=0.033
Months
3 2 2 1 1 1 1 1
9 5 5 5 5 2 1 1
25 10 7 5 4 3 1 1
20 16 12 11 10 9 2 1
F. prausnitzii SGB15323
p=0.036
Months
9 4 3 2 2 2 1 1
11 7 5 5 5 4 2 1
19 8 6 4 3 2 1 1
18 14 12 11 10 7 1 1
D
F. prausnitzii high Carnitine12:0 high
F. prausnitzii high Carnitine12:0 low
F. prausnitzii low Carnitine12:0 high
F. prausnitzii low Carnitine12:0 low
PL
F. prausnitzii SGB15316
p=0.010
Months
10 7 4 4 4 3 2 1
14 13 13 13 12 9 3 1
5 3 2 1 1 1 1 1
2 2 2 2 2 1 1 1
F. prausnitzii SGB15318
p=0.070
Months
11 6 5 4 4 3 2 1
11 9 9 9 8 6 2 1
4 4 1 1 1 1 1 1
5 5 5 5 5 4 2 1
F. prausnitzii SGB15322
p=0.082
Months
4 3 2 2 2 2 2 1
6 5 5 5 4 3 1 1
11 7 4 3 3 2 1 1
10 10 10 10 10 8 3 1
F. prausnitzii SGB15323
p<0.001
Months
7 7 5 4 4 3 2 1
6 5 5 5 5 4 2 1
8 3 1 1 1 1 1 1
10 10 10 10 9 6 2 1

Supplementary Figure 10. Acylcarnitines and *Faecalibacterium prausnitzii* segregate patients from MIND-DC trial into subsets of different prognosis.

A-B. Recurrence-free survival (RFS) analysis using the Kaplan-Meier estimator (Log-Rank (Mantel Cox) test) to assess the prognostic value of abundances of Carnitine C12:0 (A) or Carnitine C14:1 (B) and relative abundances of *F. prausnitzii* at baseline (T1) for all MIND-DC cohort (nDC+PL) (left panels) or the placebo (PL) arm only (right panels). C-D. Idem but considering only Carnitine C12:0 and various strains of *F. prausnitzii* SGB described in Supplementary Figure 2. PL, placebo; RFS, recurrence-free survival; SGB, Species-level genome bin.

Supplementary Table 1. Clinical characteristics of patients included in the translational analysis.

Translational cohort				
Variables	Overall (N=144)	nDC (N=95)	PL (N=49)	*p-value**
Center – no. (%)				
RAD	121 (84)	80 (84)	41 (84)	0.934
ISA	23 (16)	15 (16)	8 (16)	
Age, years – median (range)	57 (23-81)	57 (23-81)	56 (25-77)	0.964
Gender, male – no. (%)	83 (58)	55 (58)	28 (57)	0.931
BMI, kg/m2 – median (range)	26.4 (18.4-40.8)	26.4 (18.7-38.8)	26.2 (18.4-40.8)	0.574
Underweight or healthy weight	52 (36)	36 (38)	16 (33)	0.127
Overweight	64 (44)	37 (39)	27 (55)	
Obesity	28 (19)	22 (23)	6 (12)	
ECOG performance status – no. (%)				
0	133 (92)	90 (95)	43 (88)	0.135
1	11 (8)	5 (5)	6 (12)	
Stage – no. (%)				
IIIB	85 (59)	56 (59)	29 (59)	0.978
IIIC	59 (41)	39 (41)	20 (41)	
T staging (AJCC version 7)				
0	13 (9)	10 (11)	3 (6)	
1	15 (11)	11 (12)	4 (8)	
2	45 (32)	32 (35)	13 (27)	0.364
3	34 (24)	19 (21)	15 (31)	
4	34 (24)	20 (22)	14 (29)	
Missing	3	3	0	
N staging (AJCC version 7)				

1	58 (40)	42 (44)	16 (33)	0.364
2	39 (27)	23 (24)	16 (33)	
3	47 (33)	30 (32)	17 (35)	
Number of injections received – median (range)	6 (0-9)	5 (0-9)	6 (1-9)	0.636
Time between surgery and 1st injection, days – median (range)	98 (48-170)	99 (48-170)	97 (55-113)	0.535
≤90 days	42 (29)	26 (27)	16 (33)	0.236
>90 days	97 (67)	64 (67)	33 (67)	
Did not receive any injection	5 (3)	5 (5)	0 (0)	
Comedications at baseline, yes – no. (%)	97 (67)	64 (67)	33 (67)	0.998
Antibiotics	1 (1)	1 (1)	0 (0)	
Psychotropic drugs	14 (10)	9 (9)	5 (10)	
Metformin	4 (3)	2 (2)	2 (4)	
Antihypertensive agents	34 (24)	21 (22)	13 (27)	
Proton pump inhibitors	15 (10)	11 (12)	4 (8)	
Lipid lowering agents	22 (15)	13 (14)	9 (18)	
Metabolomics – no. (%)	144 (100)	95 (100)	49 (100)	-
Metagenomics – no. (%)	92 (64)	60 (63)	32 (65)	-

BMI: body mass index; nDC: natural dendritic cell arm; PL: placebo arm; GI: gastrointestinal; NSAID: non-steroidal anti-inflammatory drugs
*Chi-square test for contigency data, Kruskal-Wallis test for continuous data (Age).

Supplementary Table 2. Patient accrual for each omics and treatment arm in the MIND-DC ancillary study.

	nDC		PL	
	MB (n=95)	MG (n=57)	MB (n=49)	MG (n=31)
2Y-recurrence – n (%)	59 (63)	38 (67)	26 (53)	15 (48)
No 2Y-recurrence – n (%)	35 (37)	19 (33)	23 (47)	16 (52)
2Y-recurrence not reached – n	1			
2Y-death – n (%)	15 (16)	8 (14)	4 (8)	1 (3)
No 2Y-death – n (%)	79 (84)	49 (86)	45 (92)	30 (97)
2Y-death not reached – n	1			

nDC: natural dendritic cells arm ; PL: placebo arm ; MB: metabolomics ; MG: metagenomics

Supplementary Table 3. Comedications and flu-like symptoms between T1 and T2.

Translational cohort				
Variables	Overall (N=144)	nDC (N=95)	PL (N=49)	*p-value**
Started new comedications, yes – no. (%)	17 (12)	12 (13)	5 (10)	0.669
Antibiotics	4 (3)	4 (4)	0 (0)	
Systemic corticosteroids	1 (1)	1 (1)	0 (0)	
Antipyretic, anti-inflammatory, and analgesic agents (NSAID, opioids and acetaminophen)	8 (6)	5 (5)	3 (6)	
Antihypertensive agents	4 (3)	2 (2)	2 (4)	
GI drugs	3 (2)	2 (2)	1 (2)	
Others (vitamins, electrolytes, inhalatory drugs)	5 (3)	4 (4)	1 (2)	
Flu-like symptoms, yes – no. (%)	28 (19)	23 (24)	5 (10)	0.044

nDC: natural dendritic cell arm; PL: Placebo arm; GI: gastrointestinal; NSAID: non-steroidal anti-inflammatory drugs

*Chi-square test
