# Supplementary material for: Influence of microbiota-associated metabolic reprogramming on clinical outcome in patients with melanoma from the randomized adjuvant dendritic cell-based MIND-DC trial
Source: Nat Commun. 2024 Feb 23;15:1633. doi: 10.1038/s41467-024-45357-1 (PMC10891084; doi:10.1038/s41467-024-45357-1)
Supplement: Supplementary file 3 — Description of Additional Supplementary Files [file 41467_2024_45357_MOESM3_ESM.pdf]

Description of Additional Supplementary Files

Supplementary Data 1. Linear model coefficients (MaAsLin2, coefficient) for microbial SGBs that are found associated after arcsine square root (arcsin-sqrt) transformation (AST) with outcome overall (recurrence at 2 years, Figure 1).

Supplementary Data 2. Linear model coefficients (MaAsLin2, coefficient) for microbial SGBs that are found associated after arcsine square root (arcsin-sqrt) transformation (AST) with outcome (recurrence at 2 years) according to treatment arm (Supplementary Figure 1).

Supplementary Data 3. Linear models between treatment arms (nDC versus PL, Figure 3) performed via MaAsLin2 at T1.

Supplementary Data 4. Comparisons of metabolites monitored by targeted metabolomics inbetween T1 versus T2 according to treatment arm.
