# Supplementary material for: Influence of microbiota-associated metabolic reprogramming on clinical outcome in patients with melanoma from the randomized adjuvant dendritic cell-based MIND-DC trial
Source: Nat Commun. 2024 Feb 23;15:1633. doi: 10.1038/s41467-024-45357-1 (PMC10891084; doi:10.1038/s41467-024-45357-1)
Supplement: Supplementary file 9 — Reporting Summary [file 41467_2024_45357_MOESM9_ESM.pdf]

nature portfolio

Corresponding author(s): Laurence Zitvogel

Last updated by author(s): Jan 19, 2024

Reporting Summary

Nature Portfolio wishes to improve the reproducibility of the work that we publish. This form provides structure for consistency and transparency in reporting. For further information on Nature Portfolio policies, see our Editorial Policies and the Editorial Policy Checklist.

Statistics

For all statistical analyses, confirm that the following items are present in the figure legend, table legend, main text, or Methods section.

n/a	Confirmed	
☐	☒	The exact sample size (n) for each experimental group/condition, given as a discrete number and unit of measurement
☐	☒	A statement on whether measurements were taken from distinct samples or whether the same sample was measured repeatedly
☐	☒	The statistical test(s) used AND whether they are one- or two-sided<br>*Only common tests should be described solely by name; describe more complex techniques in the Methods section.*
☐	☒	A description of all covariates tested
☐	☒	A description of any assumptions or corrections, such as tests of normality and adjustment for multiple comparisons
☐	☒	A full description of the statistical parameters including central tendency (e.g. means) or other basic estimates (e.g. regression coefficient) AND variation (e.g. standard deviation) or associated estimates of uncertainty (e.g. confidence intervals)
☐	☒	For null hypothesis testing, the test statistic (e.g. F, t, r) with confidence intervals, effect sizes, degrees of freedom and P value noted<br>*Give P values as exact values whenever suitable.*
☒	☐	For Bayesian analysis, information on the choice of priors and Markov chain Monte Carlo settings
☒	☐	For hierarchical and complex designs, identification of the appropriate level for tests and full reporting of outcomes
☐	☒	Estimates of effect sizes (e.g. Cohen's d, Pearson's r), indicating how they were calculated

Our web collection on statistics for biologists contains articles on many of the points above.

Software and code

Policy information about availability of computer code

Data collection

The clinical trial data was collected and managed using the CASTOR electronic data capture platform (castoredc.com), and exported from CASTOR to CSV files for further processing.

Collection of stool and blood samples. Stool samples were prospectively collected at different time points (T1: pre-treatment, T2: 4 weeks after treatment start) at each center following the International Human Microbiome Standards (IHMS) guidelines. Both T1 and T2 samples were considered for this analysis. Blood samples were collected at the same timepoints.

Metagenomics analysis of patient stools. Overall, 185 fecal samples from 93 patients were sequenced with whole genome sequencing technology. Twelve samples did not pass internal control and were excluded from the analysis. Aliquots of stool samples were stored with DNA/RNA Shield Buffer (Zymo) at –20 °C until use. DNA was extracted from aliquots of fecal samples using the DNeasy PowerSoil Pro Kit (Qiagen) following the manufacturer's instructions. Sequencing libraries were prepared using the Illumina® DNA Prep, (M) Tagmentation kit (Illumina), following the manufacturer's guidelines. A cleaning step on the pool with 0.7x Agencourt AMPure XP beads was implemented. Sequencing was performed on a Novaseq600 S4 flow cell (Illumina) at the internal sequencing facility at University of Trento, Trento, Italy. Raw sequenced reads were QCed using the pipeline available at https://github.com/SegataLab/preprocessing. Briefly, low-quality reads (Q<20), short reads (<75bp), and reads with at least 2 ambiguous bases were discarded. Then, host DNA contaminants were removed (hg19 and phiX174 Illumina spike-in). We then obtained an average of 48 million reads per sample. For each metagenome we profiled the taxonomic and functional potential compositions with MetaPhlAn-4 33 and HUMAnN-3.6 73, respectively.

Metabolomics analysis
Serum sample preparation and widely targeted detection by LC-MS. Fifty (50) µl of collected sera were mixed with 500 µl of ice-cold

extraction mixture (methanol/water, 9/1, -20°C, with labelled internal standard). To facilitate endogenous metabolites extraction, samples were then completely homogenized (vortexed 5 minutes at 2500 rpm) and then centrifuged (10 min at 15000 g, 4°C). Supernatants were collected and several fractions were split to be analyzed by different Liquid chromatography coupled with mass spectrometers (LC/MS) 78. Polyamines and biliary acids analysis were performed by LC-MS/MS with a 1260 UHPLC (Ultra-High Performance Liquid Chromatography) (Agilent Technologies) coupled to a QQQ 6410 (Agilent Technologies) and were previously described 79. Pseudo-targeted analysis by UHPLC-HRAM (Ultra-High Performance Liquid Chromatography – High Resolution Accurate Mass) was performed on a U3000 (Dionex) / Orbitrap q-Exactive (Thermo) coupling, previously described 79,80. All targeted treated data were merged and cleaned with a dedicated R (version 4.0) package (@Github/Kroemerlab/GRMeta).

Data analysis

Metagenomics analysis of patient stools. For alpha and beta-diversity, we computed the per-sample Shannon index 74 and the between-samples Bray-Curtis dissimilarities using the implementation available in the Vegan R package 75. Differences in the distributions of alpha and beta diversity for samples collected at diagnosis with respect to recurrence at 2 years were then evaluated using Wilcoxon rank sum test 76. To test for differential abundance according to recurrence, recurrence at 2 years and treatment, we fitted a generalized linear model for each microbiome feature via the MaAsLin2 R package 77. Microbial features are first AST or CLR transformed. Adjusted P-values (Q) are computed via the Benjamini-Hochberg procedure to control for False Discovery Rate (FDR). Prevalence threshold in the differential abundance analysis was set in order to guarantee a minimum number of positive samples in each comparison. In particular, when testing for 2yR and treatment at baseline, we considered a prevalence threshold of 10%, while 30% was used when testing for 2yR, considering each treatment arm independently. We tested for differential abundance SGBs between T1 and T2 via a Wilcoxon signed-rank test considering each treatment arm and 2y-noR/2y-R combination independently. Only SGBs that were present in at least 10 samples in one of the time points are considered in this analysis.

Metabolomics analysis
Data processing and statistical analysis. Raw data were preprocessed and analyzed with R using the GRMeta package (@Github/Kroemerlab/GRMeta). This software included statistical analysis using a multivariate method approach, as PCA, Heatmap and data visualization, as volcano plots. Area intensity levels were corrected with a quality control pooled sample-based algorithm and normalized area were then log2-transformed prior to heatmaps, boxplots and volcano plots visualizations. A total of 152 metabolites were finally analyzed for serum samples at T1 and at T2. The best significant metabolites were presented with boxplots with metabolite levels log scaled. Mann-Whitney U-test with no adjustment were conducted on data gathered by two groups on processed data with R. In cases when data treatments were performed on more than two groups of patients, Kruskal-Wallis test followed by a Dunn's test with no adjustment were used on processed data. Pearson correlation analysis was applied on log2 transformed data from metabolite normalized profiles and relative abundances of F. prausnitzii SGB15318 and SGB15322. Relevant metabolites correlating with F. prausnitzii SGBs were selected and analyzed by enrichment functional analysis with Metaboanalyst (https://www.metaboanalyst.ca) using the KEGG Database38 for significant metabolites annotation and visualization.

Statistical analysis
Data analyses were performed with the Prism 10 (GraphPad, San Diego, CA, USA) and the R software. Prism always reports p-values to four decimal places. The prevalence of MGS was calculated using microbial relative abundances (MetaPhlAn-4) and considered absent if relative abundance equal to 0 and present if relative abundance superior to 0. Chi-square test was used for comparison of unpaired groups, considered significant at $p<0.05$. For MGS, two groups of patients were defined by reference values of relative abundances (MetaPhlAn-4) from publicly available HV cohort 42: high if > median and low if < median of the MGS relative abundance from HV. For key metabolites, two groups of patients were defined by its abundance median in the overall MIND-DC cohort: high if > median and low if < median. RFS analysis were performed using KM estimator. As the analysis of compositional data can lead to misleading results due to spurious correlation, we used the CLR transform to project the MGS relative abundances from the simplex to the more usual Euclidean space using the clr function of the compositions R package.
Longitudinal analysis. The analysis of the metabolite or CLR transformed microbial SGB evolution between T1, M_T1, and T2, M_T2, was performed using the following linear regression adjusted for the clinical covariables X (age, sex, BMI, stage, and ECOG-PS), considering M_T2 as response and M_T1 as offset: M_T2=M_T1+β_0+β_DC+βX. The intercept β_0 represents evolution of M (M_T2-M_T1) in the placebo arm, and β_DC represents the impact of the DC (i.e. the difference of the evolution between the two arms). The Wald test p-values of β_0 and β_DC were provided and considered as statistically significant when $p<0.05$. The evolution in the DC arm was estimated by β_0 reversing the arm reference (i.e. from the model M_T2=M_T1+β_0+β_PL+βX).
Machine Learning. All ML models were developed using R. Feature selection and prediction were based alternatively on two different outcomes: the 2Y-R and treatment arm (nDC versus PL arms). For each type of outcome, three datasets per omic (clinical features only, MB, MGS, or MB and MGS) were defined from the timepoint that was used: T1, T2, and T2-T1/T1 (pre treatment + evolution until T2 i.e. T2-T1) and a third dataset constructed by joining T2-T1 and T1 values. For each of these 18 models, clinical features were always included in both feature selection and prediction phases. For each model, clinical features were always included in both feature selection and prediction phases. The ML pipeline is based on a first step of feature selection using Boruta feature selection algorithm based on XGboost (eXtreme Gradient Boosting) algorithm 81 to identify most relevant features among clinical and biological markers (both in metabolomics or and metagenomics). A second step consisted in re-training the model from the subset of selected features re-including clinical variables in order to control potential confounding bias. A last fit of the model using selected biomarkers was applied on our training data, enabling the model to prepare for future label predictions on new data. For the multi-omics model, we performed a second step of feature selection from the metabolites and MGS selected in each omics in the first step (+ clinical variables) to obtain our final model. Missing values of metabolites were imputed using the multiple imputation by chained equations (MICE) method using the mice R package 82. We performed 50 imputations with 50 iterations to capture the uncertainty of the imputation procedure. The feature selection procedure described above was repeated using the 50 imputed datasets, and the metabolites selected in more than 75% of these 50 iterations were retained for the final step (assuming that they are robust to the randomness of the imputation). A single last imputation was performed to retrain the model in the final step. The model explainer of the different final model was based on the SHAP (Shapley Additive exPlanations) analysis, which is a visualization tool based on the following construction: SHAP values are weights associated to features for each patient, positive when the value of a marker for this patient tends to increase the prediction as class 1 (2Y-R), negative otherwise (2Y-noR). The more the absolute value of SHAP value increases, the more the feature is likely to impact the prediction (as class 1 if SHAP_value>0, class 0 otherwise). SHAP values are thus positive or negative continuous values.
Correlations between biomarkers were analyzed via the mixOmics package in R 83, using the DIABLO multiblock sPLS-DA method to display explanatory relationship between pathways and then displayed into a circosplot. The prediction performance of the whole model pipeline (feature selection to model fitting) was evaluated using the bootstrap optimism corrected area under the receiver operating characteristic (AUROC) curve. Due to the lack of external validation cohort, the optimism of the AUROC was corrected using the 0.632+ bootstrap method 84. The confidence interval of these optimism corrected AUROC was obtained using two-stage bootstrap methods proposed by Noma et al 85.

(50 internal samples, 500 external samples). The 0.632+ estimators are displayed on each figure.

For manuscripts utilizing custom algorithms or software that are central to the research but not yet described in published literature, software must be made available to editors and reviewers. We strongly encourage code deposition in a community repository (e.g. GitHub). See the Nature Portfolio guidelines for submitting code & software for further information.

Data

Policy information about availability of data

All manuscripts must include a data availability statement. This statement should provide the following information, where applicable:

- Accession codes, unique identifiers, or web links for publicly available datasets
- A description of any restrictions on data availability
- For clinical datasets or third party data, please ensure that the statement adheres to our policy

The Metagenomics data generated in this study have been deposited in the bioprojects database under accession code PRJEB66197. The Metabolomics data generated in this study have been deposited in the Mendeley Data database under accession code DOI 10.17632/nzb653783h.1 [https://data.mendeley.com/datasets/nzb653783h/1] (Suissa, D. Microbiota-associated metabolic reprograming influenced clinical outcome in the randomized dendritic cell-based clinical trial in stage III melanoma. (2023) doi:10.17632/NZB653783H.1.). The remaining data are available within the Article, Supplementary Information or Source Data file. Further individual participant clinical trial data are available under restricted access for privacy and ethical restrictions, access can be obtained by contacting the corresponding author of the companion paper (Bol KF et al., Adjuvant dendritic cell therapy in stage IIIB/C melanoma: the MIND-DC randomized phase III trial. Nat Commun (2024) In Press.) (Dr. I. Jolanda M. de Vries, e-mail address Jolanda.deVries@radboudumc.nl). Data requests will be reviewed by the principal investigators of the trial. Any data and materials that can be shared will require approval from the Institutional Review Board and a data or material transfer agreement.

Research involving human participants, their data, or biological material

Policy information about studies with human participants or human data. See also policy information about sex, gender (identity/presentation), and sexual orientation and race, ethnicity and racism.

Reporting on sex and gender	Sex and gender were not considered in the study design. Sex was collected per protocol detailed in the companion article (NCOMMS-23-20360A). Sex data was included in analysis that adjusted for the clinical covariables.
Reporting on race, ethnicity, or other socially relevant groupings	Not reported
Population characteristics	Relevant patient characteristics are described in Supplementary Table 1 and companion article (NCOMMS-23-20360A).
Recruitment	Study was open to all patients with stage III melanoma in the Netherlands and could be reffered from all centers in the Netherlands. Patients were referred by oncological surgeons to the Radboudumc. No selection was made.
Ethics oversight	The MIND-DC trial (NCT02993315) complies with all relevant ethical regulations (Dutch Central Committee on Research Involving Human Subjects). The study design and conduct complied with all relevant regulations regarding the use of human study participants and was conducted in accordance with the criteria set by the Declaration of Helsinki. Written informed consent was obtained from all patients.

Note that full information on the approval of the study protocol must also be provided in the manuscript.

Field-specific reporting

Please select the one below that is the best fit for your research. If you are not sure, read the appropriate sections before making your selection.

[x] Life sciences [ ] Behavioural & social sciences [ ] Ecological, evolutionary & environmental sciences

For a reference copy of the document with all sections, see nature.com/documents/nr-reporting-summary-flat.pdf

Life sciences study design

All studies must disclose on these points even when the disclosure is negative.

Sample size	To detect an improvement in the 2-year RFS rate from an estimated 50% to 70%, with a power of 80% and two-sided α level of 0.05, we planned to randomly assign 210 patients (companion article NCOMMS-23-20360A). In this paper, all patients with available stool and serum samples submitted to translational research were included.
Data exclusions	One patient was excluded due to insufficient follow-up to avoid censoring bias using binary classifiers.
Replication	Not applicable: Survival data is not replicable.
Randomization	Patients were randomly assigned (2:1) to receive nDC therapy or placebo. Central randomisation was based on a minimisation technique as described by Pockock et al. Patients were stratified by disease stage (IIIB vs IIIC), adjuvant radiotherapy (yes vs no), BRAFV600 mutation status (BRAF mutant vs BRAF wildtype), and HLA-type (HLA-A02:01 negative vs HLA-A02:01 positive).

Blinding	Patients treatment allocation was masked for patients and clinical investigators. Only laboratory personnel, pharmacists, and statisticians were aware of group assignment.

Reporting for specific materials, systems and methods

We require information from authors about some types of materials, experimental systems and methods used in many studies. Here, indicate whether each material, system or method listed is relevant to your study. If you are not sure if a list item applies to your research, read the appropriate section before selecting a response.

Materials & experimental systems

n/a	Involved in the study	
☒	☐	Antibodies
☒	☐	Eukaryotic cell lines
☒	☐	Palaeontology and archaeology
☒	☐	Animals and other organisms
☐	☒	Clinical data
☒	☐	Dual use research of concern
☒	☐	Plants

Methods

n/a	Involved in the study	
☒	☐	ChIP-seq
☒	☐	Flow cytometry
☒	☐	MRI-based neuroimaging

Clinical data

Policy information about clinical studies

All manuscripts should comply with the ICMJEguidelines for publication of clinical research and a completedCONSORT checklist must be included with all submissions.

Clinical trial registration	This trial is registered with EudraCT, number 2015-002531-29, and ClinicalTrials.gov, number NCT02993315
Study protocol	Accessible (submitted with companion manuscript).
Data collection	The MIND-DC study is a double-blind, randomised, placebo-controlled phase 3 study performed in two centers in the Netherlands (Radboud university medical center, Nijmegen and Isala, Zwolle).<br>Patient recruiment: Between December 2016 and November 2018.<br>Data collection: ongoing (5 years follow-up).<br>Stool samples were prospectively collected at different time points (T1: pre-treatment, T2: 4 weeks after treatment start) at each center following the International Human Microbiome Standards (IHMS) guidelines. Both T1 and T2 samples were considered for this analysis. Blood samples were collected at the same timepoints.
Outcomes	The primary endpoint was the 2-year RFS rate, defined as the percentage of patients who are alive and without recurrence of disease two years after randomization, compared to treatment with matching placebo. Patients were planned to be assessed for recurrence of disease every 3 months during the first 2 years and every 6 months thereafter up to 5 years. Disease assessment consisted of physical examination and CT scans. Other imaging techniques were used as clinically indicated. Recurrent disease was histologically confirmed, whenever possible.<br>Secondary endpoints were median recurrence-free survival (RFS), 2-year and median OS, adverse event (AE) profile, and immunological response. Adverse events were recorded using the Common Toxicity Criteria for Adverse Events version 4.03 up to 30 days after the last administration of study treatment or start of another cancer therapy, whichever occurred first. Serious adverse events believed to be related to the study treatment were still recorded after this period. Apheresis-related AEs are defined as all related AEs within one week of apheresis. AEs are considered related to the treatment/apheresis if the event was recorded as possible, probable, or definite related to the apheresis procedure by the treating physician.<br>The Kaplan-Meier method was used to estimate median RFS and OS distributions and the 90% CI of these estimates. A comparison between the groups was made using the log-rank test. Hazard ratios were estimated with a Cox proportional hazards model, stratified by stage of the disease, adjuvant radiotherapy, BRAF mutation status, and HLA-type. RFS was defined as the time between randomization and the date of first recurrence (local, regional, or distant metastasis) or death, whichever occurred first. P values for differences between fractions (such as the fraction of patients showing an immune response in the nDC treatment group versus the control group) were calculated by means of the chi-square test. When events had not occurred, survival was censored at the date of last follow-up. We calculated median follow-up using the inverse Kaplan-Meier method. Efficacy analysis was performed on the intention-to-treat population, defined as all eligible patients assessed in the group they were allocated by randomisation. The safety population consisted of all patients who at least started apheresis.

Plants

Seed stocks	Not applicable
Novel plant genotypes	Not applicable
Authentication	Not applicable
